# The burden of congenital rubella syndrome in the Philippines: results from a retrospective assessment

**DOI:** 10.5365/wpsar.2017.8.1.006

**Published:** 2017-06-13

**Authors:** Anna Lena Lopez, Peter Francis Raguindin, Jose Jonas del Rosario, Ramon V Najarro, Eleanor Du, Josephine Aldaba, Aida M Salonga, Andrea Kristina Monzon-Pajarillo, Alvina Pauline Santiago, Alan C Ou, Maria Joyce Ducusin

**Affiliations:** a Institute of Child Health and Human Development, National Institutes of Health, University of the Philippines Manila, Philippines.; b Philippine Children’s Medical Center, Quezon City, Philippines.; c Department of Pediatrics, College of Medicine, University of the Philippines Manila, Philippines.; d Department of Pediatrics, Vicente Sotto Memorial Medical Center, Cebu City, Philippines.; e Department of Pediatrics, Southern Philippines Medical Center, Davao City, Philippines.; f Department of Ophthalmology, College of Medicine, University of the Philippines Manila, Philippines.; g World Health Organization Representative’s Office, Manila, Philippines.; h Centers for Disease Control and Prevention, Global Immunization Division, Atlanta, Georgia, USA.; i Department of Health, Philippines.

## Abstract

**Introduction:**

In line with the regional aim of eliminating rubella and congenital rubella syndrome (CRS), phased introduction of rubella-containing vaccines (RCV) in the Philippines’ routine immunization programme began in 2010. We estimated the burden of CRS in the country before widespread nationwide programmatic RCV use.

**Methods:**

We performed a retrospective chart review in four tertiary hospitals. Children born between 1 January 2009 and 31 December 2014 and identified as possible CRS cases based on the presence of one or more potential manifestations of CRS documented in hospital or clinic charts were reviewed. Cases that met the clinical case definition of CRS were classified as either confirmed (with laboratory confirmation) or probable (without laboratory confirmation). Cases that did not fulfil the criteria for either confirmed or probable CRS were excluded from the analysis.

**Results:**

We identified 18 confirmed and 201 probable cases in this review. Depending on the hospital, the estimated incidence of CRS ranged from 30 to 233 cases per 100 000 live births. The estimated national burden of CRS was 20 to 31 cases per 100 000 annually.

**Discussion:**

This is the first attempt to assess the national CRS burden using in-country hospital data in the Philippines. Prospective surveillance for CRS and further strengthening of the ongoing measles-rubella surveillance are necessary to establish accurate estimates of the burden of CRS and the impact of programmatic RCV use in the future.

## Introduction

Rubella, also known as German measles, is an exanthematous disease that commonly causes mild fever and rash that begins on the face and gradually spreads to the neck, trunk and extremities. While most infections are mild, infection in a pregnant woman may cause devastating fetal malformations and may result in stillbirths, miscarriage or a pattern of birth defects known as congenital rubella syndrome (CRS). (*1*-*3*)

The use of effective rubella-containing vaccines (RCV) has resulted in significant reductions in the incidence of rubella and CRS in countries that have included rubella vaccines in their national immunization programmes. In 2015, it was announced that the countries in the World Health Organization (WHO) Region of the Americas had eliminated endemic transmission of rubella and CRS. (*4*) Before routine rubella vaccination, the incidence of CRS worldwide ranged from 10 to 20 cases per 100 000 live births to 80 to 400 cases per 100 000 live births during intra-epidemic and epidemic periods, respectively. (*3*, *5*-*7*) Globally, it is estimated that there were 105 391 cases of CRS in 2010, representing a decline of 11.6% from 1996. (*8*) In the WHO Global Vaccine Action Plan 2011–2020, a goal to eliminate both measles and rubella in at least five regions of the WHO was established. (*9*) In October 2014, the  WHO Regional Committee for the Western Pacific Region included rubella elimination plus CRS prevention as one of eight regional immunization goals specified by the Regional Framework for Implementation of the Global Vaccine Action Plan in the Western Pacific. (*10*) To support this goal, the Technical Advisory Group on Immunization and Vaccine Preventable Diseases in the Western Pacific Region recommended enhancing surveillance activities for rubella and CRS with case detection and thorough outbreak investigations as well as appropriate case management and vaccination of susceptible contacts. (*11*) In the Philippines, rubella surveillance is conducted as part of measles surveillance. No CRS surveillance currently exists anywhere in the Philippines.

In the Philippines, a pilot project introduced RCV in five of the 18 regions of the country in 2009. In 2010, RCV was incorporated into the national routine immunization programme targeting children aged 12–15 months with the combined measles-mumps-rubella (MMR) vaccine. Children up to the age of 95 months were additionally covered by a national measles and rubella supplemental immunization campaign in 2011. (*12*) Coverage for MMR gradually rose from 31% in 2011 to 38% in 2012–2013, and 64% in 2014; it was 62% in 2015. MMR coverage remained low due to vaccine stock-outs in 2013 and 2015 and delayed reporting from the 18 regions. (*13*) To date, women of childbearing age have not been targeted systematically for rubella vaccination in the Philippines.

We aimed to estimate the burden of CRS in the country through a retrospective chart review to provide a baseline before widespread introduction of rubella vaccines. This information is important for evaluating the impact of the introduction of RCV into the immunization programme.

## Methods

**Fig. 1:**
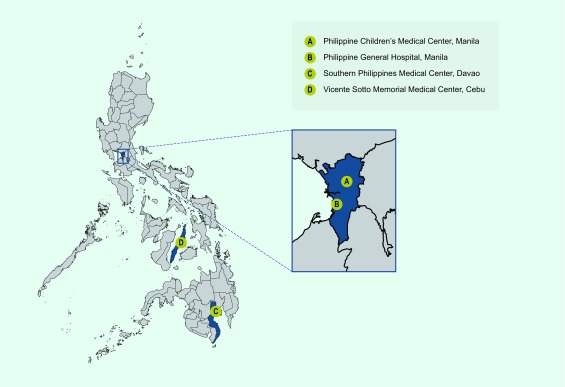
Map of the Philippines with location of the study hospitals

We conducted a retrospective review of hospital records in four large hospitals in the country. These hospitals, which are public, tertiary training hospitals equipped with subspecialists capable of managing CRS, are known to have the highest annual CRS consultations. They were selected based on their large catchment area that encompasses the three main island groups of the Philippines as well as their ability to provide care to CRS cases. Two of the hospitals were in Metro Manila in the most populated island of Luzon (Philippine General Hospital, PGH, in the City of Manila, and Philippine Children’s Medical Center, PCMC, in Quezon City), one in Cebu City in the Visayas (Vicente Sotto Memorial Medical Center, VSMMC) and one in Davao City in Mindanao (Southern Philippines Medical Center, SPMC) (**Fig. 1**).

### Records review and case classification

The following patients were included in the review: children born between 1 January 2009 and 31 December 2014 who were hospitalized or received outpatient care at one of the study sites from 1 January 2009 until 31 December 2014 with:

- documented positive rubella immunoglobulin M (IgM) laboratory test result; OR
- International Classification of Disease (ICD)-9 (*14*) or ICD-10 (*15*) discharge code consistent with one or more manifestation(s) of CRS; ICD-9/ICD-10 codes used in the chart review were:
  1. congenital rubella syndrome (771.0/P35);
  2. cataracts (743.3/Q12);
  3. congenital glaucoma (743.2/Q15-H40);
  4. deafness and hearing impairment (389.1/H90);
  5. congenital heart disease (745–747/Q20-Q26);
  6. dermal erythropoiesis (759.89/P83.8);
  7. microcephaly (742.1/Q02); OR
- written documentation in the medical record of one or more manifestation(s) of CRS using the following diagnostic keywords:
  1. cardiac—congenital heart disease (CHD);
  2. patent ductus arteriosus (PDA);
  3. peripheral pulmonary artery stenosis;
  4. congenital cardiopathy;
  5. ventricular septal defect;
  6. ophthalmologic: cataract, microphthalmia, glaucoma, pigmentary retinopathy;
  7. auditory: deafness, hearing loss/hearing impairment;
  8. dermatologic: purpura, “blueberry muffin rash”; and
  9. others: microcephaly, mental retardation, developmental delay, neonatal jaundice, hepatosplenomegaly, meningoencephalitis, radiolucent bone disease, “rule out ToRCH infection,” congenital rubella syndrome or congenital rubella infection (including “suspected CRS” or “rule out congenital rubella”).

We excluded the following in our review: infants < 2500 g with isolated PDA or isolated microcephaly and no other signs of CRS, documented negative rubella-specific IgG test for the child, documented positive laboratory test for other potential etiology of CRS manifestation (e.g. positive cytomegalovirus or toxoplasmosis test) in the absence of a positive rubella laboratory test and not a resident of the Philippines.

Charts were retrieved from all eligible cases. Information collected from the charts included hospital location; patient’s province and region of residence; location of birth, maternal and infant demographics; infant’s clinical signs and symptoms; maternal history; and laboratory tests performed. Data were collected on standard forms and entered securely into an electronic database using Epi Info 7 (Centers for Disease Control and Prevention, Atlanta, Georgia, USA. Participants were coded using a unique surveillance identification number.

### Data analysis

Data analysis was performed using Epi Info 7. We used the case definition from WHO surveillance standards (*16*, *17*) to classify the identified cases (**Box 1**). Estimated annual incidence rates were calculated using different methods. First, we computed hospital-specific incidence including only babies who were born at PGH, SPMC or VSMMC in the analysis. Since few deliveries occurred in PCMC, incidence rate for this hospital was not calculated. The numerator was the respective number of probable or confirmed CRS cases in one of the three study sites and the denominator was the number of live births in the same hospitals from 1 January 2009 to 31 December 2014. To calculate the national incidence rates, we used the method previously used by Bloom, et al. using cataract detection in Morocco to calculate the national burden (*19*) with the following formula:

**Figure:**
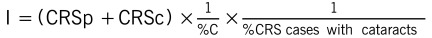


Where I = incidence, CRSp = probable CRS cases, CRSc = confirmed CRS cases,%C = percentage of overall cataract care provided at three participating hospitals, and%CRS cases with cataract = CRS cases with cataracts based on previous literature.

Based on previous studies, 16–25% of CRS cases have cataracts. (*20*, *21*) For the national incidence estimation, we obtained the proportion of cataract care provided by each participating hospital by using the insurance claims for ICD-10 code Q12 (congenital cataract and congenital diseases of the lens) from PhilHealth (the National Health Insurance Programme). Based on the claims from PhilHealth from 2009 to 2013, PGH, PCMC, SPMC and VSMMC accounted for 7%, 0%, 2% and 1% of all cataract care in the country, respectively, or 10% cumulatively for all hospitals. (*22*) This database included reports from both private and public hospitals in the country that managed cases of congenital cataracts.

**Figure:**
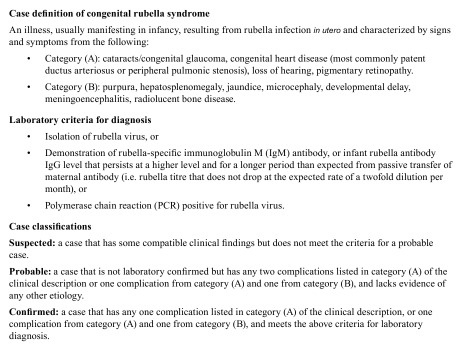


This study was reviewed and approved by the Ethics Review Committee of the WHO Regional Office for the Western Pacific (2015.8.PHL.2.EPI), and the ethical review boards of the University of the Philippines Manila (UPM-REB 2015–205–01), PCMC, VSMMC and the SPMC.

## Results

Out of 4339 unique entries identified from medical records, we identified 18 laboratory-confirmed cases and 201 probable CRS cases from the four hospitals. The majority of suspected cases came from PGH (1849), followed by PCMC (1091), SPMC (939) and VSMMC (459). Both SPMC and VSMMC had no confirmed cases due to the absence of laboratories capable of performing a rubella IgM test in either Davao City or Cebu City. Clinical manifestations of CRS were predominantly cardiac (83.3% and 86.1% among confirmed and probable cases, respectively), audiologic (50% and 33.3% among confirmed and probable cases, respectively) and ophthalmologic (27.8% and 25.4% among confirmed and probable cases, respectively). Among all confirmed and probable CRS cases, the mean age of diagnosis was 9.9 months (range: 3 days–72 months) with more cases among males (55.7%) and the mean age of mothers was 27.8 (± 5.2) years, with only 13.2% reporting rashes on prenatal hi story by recall (**Tables 1**** and ****2**). The most common cardiac presentation was patent ductus arteriosus.

**Table 1:**
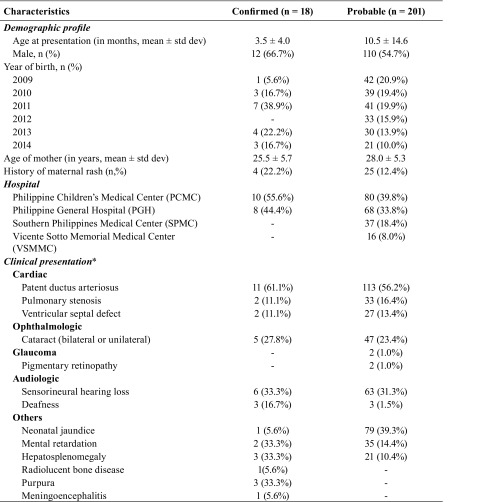
Characteristics of confirmed and probable CRS cases

* Cases may have more than one cardiac and ophthalmologic manifestation.

Figures should not be considered as part of a whole.

**Table 2:**
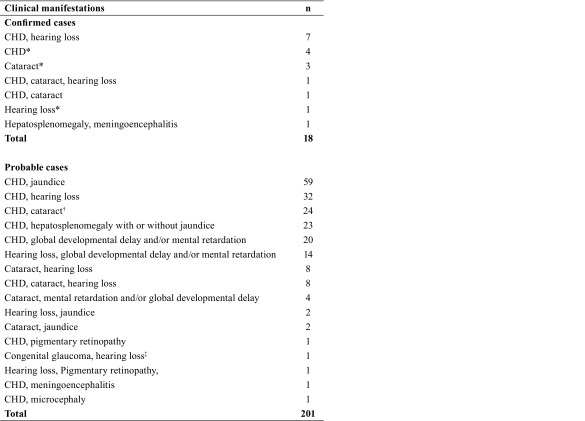
Clinical profile of confirmed and probable CRS cases

CHD = congenital heart defect

* Had other minor manifestations

^†^ Among the 24 patients, one had CHD, bilateral cataract and congenital glaucoma

^‡^ Had combined bilateral cataract and congenital glaucoma

We obtained the number of live births in PGH, VSMMC and SPMC. Using each hospital’s live births, the estimate for CRS incidence ranged from 30 to 233 cases per 100 000 live births (**Table 3**).

**Table 3:**
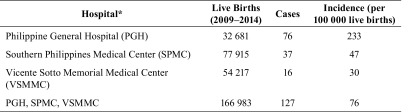
Clinical profile of confirmed and probable CRS cases

* Since few deliveries occurred in PCMC, incidence rate for this hospital was not calculated.

There were 52 cataract cases among the 219 confirmed and probable cases identified from 2009 to 2014. Based on PhilHealth claims for congenital cataracts from 2009 to 2013, PGH, PCMC, SPMC and VSMMC together accounted for 10% of all cataract cases nationwide. Thus, there were an estimated 520 diagnosed cataract cases nationally from 2009 to 2014. Using the reported live births in the country during the same period, (*23*) and adjusting by 4–6.25 times (the inverse of 16–25% of CRS cases have cataracts), then an estimated 2080 to 3250 CRS cases nationally from 2009 to 2014, or an annual incidence of 20 to 31 CRS cases per 100 000 live births.

## Discussion

We documented the occurrence of CRS in the Philippines; cardiac and ophthalmologic defects were the most common findings, similar to previous studies conducted in Sudan, (*24*) Viet Nam (*25*) and the Philippines. (*26*) Our estimates for CRS varied widely by hospital. WHO estimates that there were 150 cases of CRS per 100 000 live births in the Philippines in 2010, or about 2674 cases of CRS, much higher than estimates obtained in this review. (*27*) Previous estimates of CRS were based on modelling using rubella seroprevalence data together with the incidence of infection during gestation (*28*) or with immunization coverage in the different countries, (*8*) while this study was a retrospective assessment of CRS using admission records.

The national estimate we obtained based on cataract care is conservative. First, our review covered only a small proportion of the country and is not representative of the entire population. We conducted chart reviews in four public hospitals that were the biggest tertiary public referral centres in the country’s three major island groups and located in urbanized centres. As CRS diagnosis requires consultation with subspecialists that is typically unavailable at small hospitals, most cases should have been referred to one of these hospitals. A closer review of the data from PGH and PCMC showed that only 59% and 57%, respectively, of the patients came from Metro Manila; the rest came from other areas. But despite the four hospitals’ large catchment areas, there are more than 1800 hospitals in the Philippines. In addition, since only 40% of Philippines’ hospitals are government-owned, some patients may have sought care in the private sector. It is estima ted that 30% of the population use private fee-for-service medical care. (*29*) Second, there are differences in the hospitals included in the study. The higher incidence seen in PGH compared to SPMC and VSMMC may be due to the nature of deliveries performed at PGH. PGH is the largest training and referral hospital in the Philippines and only high-risk pregnancies are admitted; hence normal deliveries are limited at the hospital. PGH is also considered to have the most complete subspecialty services; thus patients requiring complicated case management are often transferred to this hospital. Conversely, the hospitals in Cebu and Davao did not have adequate laboratories to diagnose CRS. Subspecialty services (paediatric ophthalmology and audiology) were also inconsistently available during the inclusive dates under review. Thus, children seeking eye care and hearing tests may have sought care at private health facilities an d therefore possibly missed. With the passage of a law in 2009 that requires mandatory hearing screening of all newborns, more public facilities are able to conduct hearing testing and identify cases. Third, as in any retrospective chart review, we encountered difficulties in retrieving patient records and abstracting information from clinical sources. A significant number of medical records were missing in the archiving facilities of respective hospitals. Retrieved medical records, likewise, had incomplete documentation. The incomplete records and inaccurate coding may also result in misclassification and reduce our estimates. Fourth, we found many cases in which care from hospitals was sought late. Many children with hearing and visual impairment were seen after 5 years of age and therefore were missed in this retrospective case finding. In PGH, only 30% of children with hearing loss were referred before 1 year of age, (*30*) and CRS was the most common (36%) etiology of hearing loss in 94 patients who underwent cochlear implantation. (*31*) Fifth, the estimate on the national incidence is likely to be an underestimate due to the low utilization and coverage of PhilHealth for the lower economic strata from 2009 to 2014. Although 88% of the population were enrolled in 2015 in PhilHealth, from 2009 to 2014 PhilHealth utilization remained low. (*22*) Lastly, the phased introduction of RCV may have affected our results since RCV was initially introduced in 2009 before inclusion into the national routine immunization programme targeting children aged 12–15 months with the MMR vaccine and as supplemental immunization campaigns in children up to the age of 95 months in 2011 resulting in low RCV coverage initially but increasing coverage as the study progressed. However, by 2014, the national childhood RCV coverage was < 70% due to vaccine stock-outs and in Metro Manila, RCV coverage was < 50%. At this vaccine coverage, it is unlikely that susceptible pregnant women would benefit from herd immunity. (*32*)

Currently, women of childbearing age are not systematically targeted for rubella vaccination in the Philippines. In 2002, 15% of women in an urban antenatal clinic remained susceptible to rubella. (*26*) In the absence of vaccination, a large cohort of this population remains at risk for being infected with rubella during pregnancy. From 1 January to 22 October 2016, there were 119 laboratory-confirmed cases of rubella out of 1732 suspected measles-rubella cases captured by the Philippine Department of Health surveillance. Of these, 23% of cases were among women aged 16 to 30 years. (*33*)

To the best of our knowledge, this is the first attempt to obtain an estimate of the burden of CRS using hospital data in the Philippines. The estimates varied widely by hospital and the national estimate we obtained was substantially lower than those obtained from models. Prospective surveillance will be important to obtain the true burden of CRS in the Philippines. New CRS surveillance guidelines are now available and these will be used as the country strengthens its rubella surveillance and plans to embark on a prospective CRS surveillance. Care must be taken in choosing potential surveillance sites to obtain reliable data.
